# The Effectiveness of Hyperbaric Oxygen Therapy on Older Patients With Medication-Related Osteonecrosis of the Jaws: A Case Series

**DOI:** 10.7759/cureus.69226

**Published:** 2024-09-11

**Authors:** Taku Kimura, Keisuke Kusano, Ken-ichiro Sakata, Jun Sato, Yoshimasa Kitagawa

**Affiliations:** 1 Department of Oral Diagnosis and Medicine, Hokkaido University, Sapporo, JPN

**Keywords:** antiresorptive agents, case series, hbo therapy, medication-related osteonecrosis of the jaw, oral surgery

## Abstract

Medication-related osteonecrosis of the jaws (MRONJ) has emerged as one of the major adverse effects of antiresorptive agents in the treatment of patients with cancer and osteoporosis. MRONJ presents as a chronic inflammation of the maxillary and/or mandibular bones accompanied by necrotic bone exposure and intra-/extraoral fistula. Given the increasing number of patients with MRONJ, surgical treatment is highlighted to be significantly beneficial for those patients. However, extensive surgical treatment generally induces physiological and psychological burden on patients with MRONJ. Specifically, older patients with advanced MRONJ require further concerns about their systemic conditions. Thus, oral surgeons are obliged to consider their conditions when determining the indications for extensive surgical treatment. Recently, our department has established a novel therapeutic strategy based on hyperbaric oxygen (HBO) therapy for patients with advanced MRONJ. In this study, we report cases of three older patients with MRONJ who received the combination of conventional treatment and HBO therapy, which resulted in successful management and the avoidance of extensive surgical treatment.

## Introduction

Medication-related osteonecrosis of the jaws (MRONJ) is an intractable inflammatory disease associated mainly with androgen receptor antagonists (ARAs) in treating patients with cancer and osteoporosis [1]. The number of patients with MRONJ has been increasing worldwide, and its incidence rate depends on the medical history of the patients, including the amount and duration of ARAs, particularly bisphosphonate (BP) and anti-receptor activator of nuclear factor kappa-B ligand (RANKL) antibody, which ranges from 0.02% to 18% [1-3]. According to the position paper published by the American Association of Oral and Maxillofacial Surgeons, MRONJ stages are divided into four classes (stages 0-3) determined by clinical and radiographic findings [1]. Therapeutic strategies for patients with MRONJ include conservative treatment with oral irrigation, antimicrobial treatment, and surgical treatment [2]. Of note, surgical treatment for patients with advanced MRONJ (stages 2 and 3) offers significant benefits than conservative treatment alone [3,4]. Furthermore, radical treatment such as marginal resection and segmental resection of the jaws has shown better clinical outcomes than conservative surgery including sequestrectomy, soft tissue debridement, and saucerization [5,6].

Currently, hyperbaric oxygen (HBO) therapy has been employed widely in treating various diseases including osteoradionecrosis of the jaw (ORN), types of refractory inflammatory disease in the oral and maxillofacial region, and other medical fields such as chronic diabetic foot ulcer and severe systemic inflammation [7-10]. As for the effectiveness of HBO therapy among patients with MRONJ, this therapy is also reported to provide mild benefit when combined with conventional treatment compared with conventional treatment alone [8,9,11]. However, few studies have demonstrated the definitive effectiveness of HBO therapy among these patients, retaining its position as an adjunctive therapy. Recently, we have proved that nuclear medicine examinations including fluorodeoxyglucose-positron emission tomography (FDG-PET) and bone single-photon emission computed tomography (SPECT) are useful modalities to diagnose MRONJ and evaluate the effect of HBO therapy on patients with MRONJ [12]. Therefore, our department has recently established a therapeutic strategy based on HBO therapy for patients with advanced MRONJ. This therapeutic strategy involves HBO therapy and conservative treatment with oral irrigation, antimicrobial treatment, and/or conservative surgery.

Considering the global situation where the older population has been increasing, the number of older patients with MRONJ should also be increasing. Radical treatment with extensive invasiveness is preferred for patients with advanced MRONJ; however, older patients generally have poor surgical tolerance and several medical backgrounds, which limit the treatment options. Therefore, this study aimed to offer alternative therapeutic strategies based on HBO therapy for patients with MRONJ who are not indicated or willing to undergo radical treatment. Herein, we present cases of three older patients with advanced MRONJ that was successfully controlled by conventional treatment along with HBO therapy.

## Case presentation

Data from three patients diagnosed with MRONJ are summarized in Table 1.

**Table 1:** Background of three patients diagnosed with advanced MRONJ. *At first visit to our department. MRONJ, medication-related osteonecrosis of the jaws; BP, bisphosphonate; RANKL, receptor activator of nuclear factor kappa-B ligand

Patient	Number 1	Number 2	Number 3
Age*	81	68	87
Gender	Female	Female	Female
Medical background	Iron-deficiency anemia, hypertension, hyperlipidemia, and osteoporosis	Hypertension, type 2 diabetes mellitus, depression, and osteoporosis	Cerebral infarction, aortic incompetence, mitral incompetence, and osteoporosis
Medication, duration/month	Alendronic acid (BP), 13 months; denosumab (anti-RANKL antibody), two months	Alendronic acid (BP), 12 months; zoledronic acid (BP), 12 months	Ibandronate acid (BP), 19 months
Location	Right-sided mandibular molar	Maxillary anterior and right-sided maxillary molar and mandibular anterior	Right-sided mandibular molar
Initial clinical findings	Extensive bone exposure with extraoral fistula in the right-sided mandibular molar and swelling skin around the right-sided mandibular angle	Extensive bone exposure reigning from the maxillary anterior to the right-sided maxillary molar and mandibular anterior and swelling gingiva around the right-sided maxillary molar	Swelling and redness of the skin around the right-sided submandibular with extra-fistula
Initial radiographic findings	Osteolytic lesion with bone sequestration	Osteolytic lesion with bone sequestration	Osteolytic lesion with bone sequestration and apparent periosteal reaction
Diagnosis	Stage 3	Stage 2	Stage 3

All patients had a history of osteoporosis and were treated with BP and/or anti-RANKL antibody. In concurrence with their MRONJ treatment, each patient was referred to their family doctor to replace ARAs with active vitamin D metabolites to halt disease progression. Each patient underwent HBO therapy with a hyperbaric oxygen chamber (KHO-301B, Kawasaki Engineering, Kobe, Japan). The HBO therapy protocol in our department was as follows: compressing to 2.4 absolute atmosphere (0.146 MPa gauge pressure) at a constant rate for 15 minutes, followed by maintained pressure for 60 minutes and decompression to normal pressure for 20 minutes.

Patient 1

**Figure 1:**
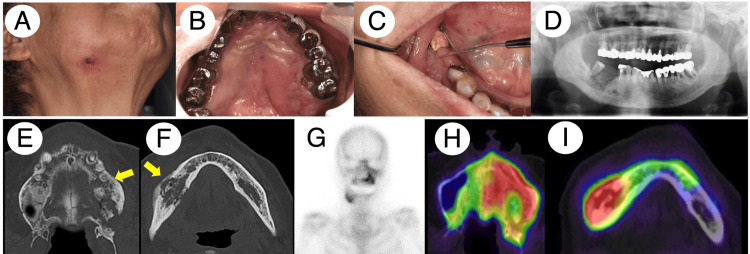
Patient 1 clinical and radiographic findings before the treatment. Extraoral examination identified a fistula in the right-sided mandibular skin (A). Intraoral examination detected bone exposure probing through intraoral fistula in the left-sided maxillary molar area and right-sided mandibular molar area (B and C). Panoramic X-ray and MDCT depicted sequestrum and abnormal sclerotic changes in the affected areas with apparent periosteal reaction in the mandibular bone (the yellow arrow indicates the signs of sequestrum in the maxillary and mandibular bones) (E and F). Bone scintigraphy and SPECT showed the increased accumulation of 99mTc-MDP/HMDP in the affected areas (G, H, and I). MDCT, multidetector computed tomography; SPECT, single-photon emission computed tomography; 99mTc, technetium-99m; MDP, methylene diphosphonate; HMDP, hydroxy-methylene diphosphonate

An 81-year-old female patient with a history of osteoporosis treated with BP for 13 months and anti-RANKL antibody for two months was referred to our department with a complaint of swelling right-sided mandibular skin. Extraoral examination revealed an extraoral fistula in the right-sided mandibular skin (Figure 1A). Intraoral examination identified bone exposure in the left-sided maxillary molar area and right-sided mandibular molar area with the surrounding gingiva exhibiting mild redness (Figure 1B, 1C). Panoramic X-ray examination detected signs of sequestrum in both the bone-exposed areas with vertical bone loss (Figure 1D). Moreover, multidetector computed tomography (MDCT) unveiled the extensive sequestrum formation in both bone-exposed areas, and the right-sided mandibular bone presents an apparent periosteal reaction (Figure 1E, 1F). Nuclear medicine modalities highlighted the bone-exposed area with a significantly increased accumulation of technetium-99m (99mTc)-methylene diphosphonate (MDP)/hydroxy-methylene diphosphonate (HMDP) in the sequestrum area by bone scintigraphy and SPECT (Figure 1G, 1H, 1I). Considering these findings, the patient was diagnosed with MRONJ stage 3 in the left-sided maxillary and right-sided mandibular bones.

**Figure 2:**
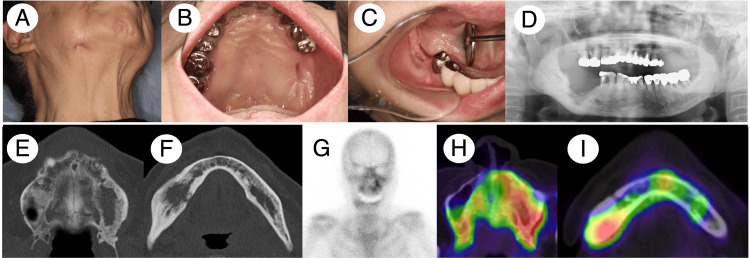
Patient 1 clinical and radiographic findings after the treatment. Extra- and intraoral examination unveiled the remaining cavity covered with granulation tissue or normal gingiva (A, B, and C). Panoramic X-ray and MDCT identified the signs of bone regeneration in the affected areas with periosteal reaction disappearance (D, E, and F). Bone scintigraphy and SPECT showed an apparent reduction of the 99mTc-MDP/HMDP accumulation in the affected areas (G, H, and I). MDCT, multidetector computed tomography; SPECT, single-photon emission computed tomography; 99mTc, technetium-99m; MDP, methylene diphosphonate; HMDP, hydroxy-methylene diphosphonate

The patient received oral irrigation regularly and antimicrobial treatment with amoxicillin (AMPC) 1,500 mg/day alone or AMPC 1,500 mg/day and clavulanic acid (CVA) 375 mg/day for 33 days in total throughout her hospitalization. Thereafter, the patient was hospitalized in our department, received preoperative HBO therapy 10 times, and underwent sequestrectomy under intravenous anesthesia. Histopathological examination identified sequestrum with granulation tissue and bacterial colonies, resulting in MRONJ as the final diagnosis. Then, the patient received postoperative HBO therapy 10 times in two weeks with AMPC for three days after the surgery and was finally discharged from our hospital, without any postoperative complications. Six months after the treatment, extra- and intraoral examinations did not reveal signs of pus drainage from the residual fistula covered with granulation tissue in the skin or normal gingiva in the oral region (Figure 2A, 2B, 2C). Panoramic X-ray and MDCT examinations revealed signs of bone regeneration in the affected mandibular and maxillary areas (Figure 2D, 2E, 2F). Additionally, the bone scintigraphy and SPECT detected the decreased accumulation of 99mTc-MDP/HMDP in the affected areas (Figure 2G, 2H, 2I). Furthermore, GI-BONE (Nihon Medi-Physics Co., Ltd., Tokyo, Japan) revealed that metabolic bone volume (MBV) declined from 18.34 to 17.46 cm^3^ in the maxillary lesion and from 32.90 to 23.91 cm^3^ in the mandibular lesion, and the maximum standardized uptake value (SUVmax) also decreased from 12.16 to 10.17 in the maxillary lesion and from 15.39 to 11.41 in the mandibular lesion. These results indicated the improvement of the MRONJ inflammatory activity.

Patient 2

**Figure 3:**
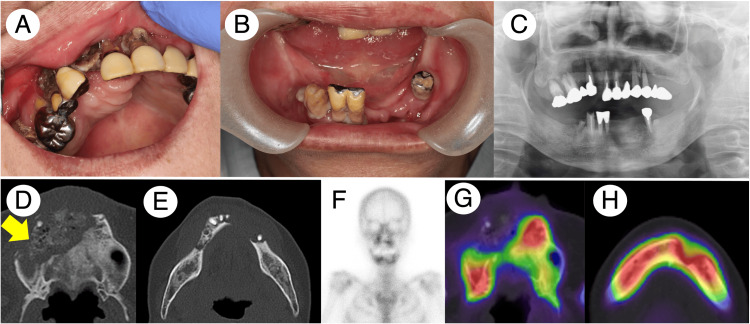
Patient 2 clinical and radiographic findings before the treatment. Intraoral examination found extensive bone exposure in both maxillary and mandibular bones (A and B). Panoramic X-ray imaging exhibited an irregular margin of the alveolar bone in both the maxillary and mandibular bones (C). MDCT revealed the extensive formation of the sequestrum and apparent sclerotic changes in the affected areas (the yellow arrow indicates the sequestrum in the maxillary bone) (D and E). Bone scintigraphy and SPECT showed the increased accumulation of 99mTc-MDP/HMDP in the affected areas (F, G, and H). MDCT, multidetector computed tomography; SPECT, single-photon emission computed tomography; 99mTc, technetium-99m; MDP, methylene diphosphonate; HMDP, hydroxy-methylene diphosphonate

A 68-year-old female patient with a history of osteoporosis treated with BP for 132 months was referred to our department for a complaint of frequent swelling in the right-sided mandibular skin and tooth loosening. Extraoral examination revealed numbness around the right-sided lower lip skin, whereas intraoral examination identified extensive bone exposure ranging from the upper anterior to the right-sided maxillary molar area, with the teeth close to the lesion presenting significant mobility, and the sporadic bone exposure was identified around the lower anterior area (Figure 3A, 3B). Panoramic X-ray examination detected extensive horizontal bone loss in both mandibular and maxillary bones (Figure 3C). In addition, MDCT unveiled the extensive sequestrum in both bone-exposed areas and apparent sclerotic changes within her mandibular alveolar bone without any signs of periosteal reaction (Figure 3D, 3E). Nuclear medicine modalities highlighted the extensive bone-exposed area with robust accumulation of 99mTc-MDP/HMDP around the sequestrum area by bone scintigraphy and SPECT (Figure 3F, 3G, 3H). Therefore, the patient was diagnosed with MRONJ stage 2 in the anterior mandibular and anterior to the right-sided maxillary bone.

**Figure 4:**
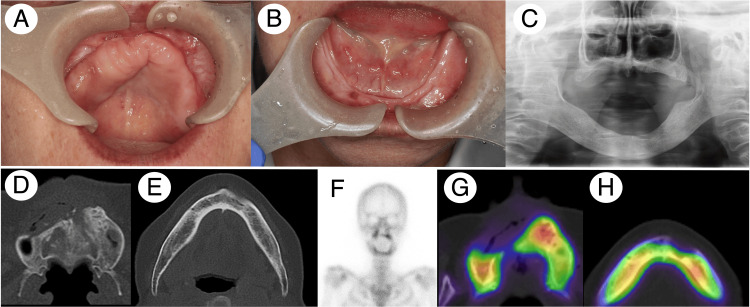
Patient 2 clinical and radiographic findings after the treatment. Intraoral examination revealed no signs of bone exposure or cavity remaining in the alveolar area (A and B). Panoramic X-ray imaging identified the smooth edge of the affected bone without any abnormal osteolytic or sclerotic findings (C, D, and E). Bone scintigraphy and SPECT showed a mild reduction of 99mTc-MDP/HMDP accumulation in the affected areas (F, G, and H). SPECT, single-photon emission computed tomography; 99mTc, technetium-99m; MDP, methylene diphosphonate; HMDP, hydroxy-methylene diphosphonate

As for the numbness around the lower lip skin, the patient received 1,500 μg/day of vitamin B12 for a month. The patient further received oral irrigation regularly and antimicrobial treatment with AMPC 1,500 mg/day and CVA 375 mg/day for 64 days in total until her hospitalization. Thereafter, the patient was hospitalized in our department, received preoperative HBO therapy 20 times, and underwent sequestrectomy and saucerization under general anesthesia. Histopathological examination identified sequestrum with granulation tissue and bacterial colonies, resulting in MRONJ as the final diagnosis. Then, the patient received postoperative HBO therapy 10 times in two weeks with AMPC for three days after the surgery and was finally discharged from the hospital without causing any postoperative complications. A week after treatment completion, an intraoral examination was performed, which identified a smooth alveolar ridge without signs of bone exposure or remaining fistula with numbness around the lower lips (Figure 4A). Panoramic X-ray and MDCT examinations revealed the smooth edges of the alveolar bones in both the maxillary and mandibular areas (Figure 4C, 4D, 4E). Moreover, the bone scintigraphy and SPECT identified a mild reduction in 99mTc-MDP/HMDP accumulation in the affected areas (Figure 4F, 4G, 4H). In addition, the GI-BONE software revealed that the MBV declined from 58.37 to 33.57 cm^3^ in the maxillary lesion and from 22.13 to 7.43 cm^3^ in the mandibular lesion. The SUVmax also decreased from 12.67 to 10.01 in the maxillary lesion and from 17.86 to 7.59 in the mandibular lesion. These results indicated the improvement of MRONJ inflammatory activity.

Patient 3

**Figure 5:**
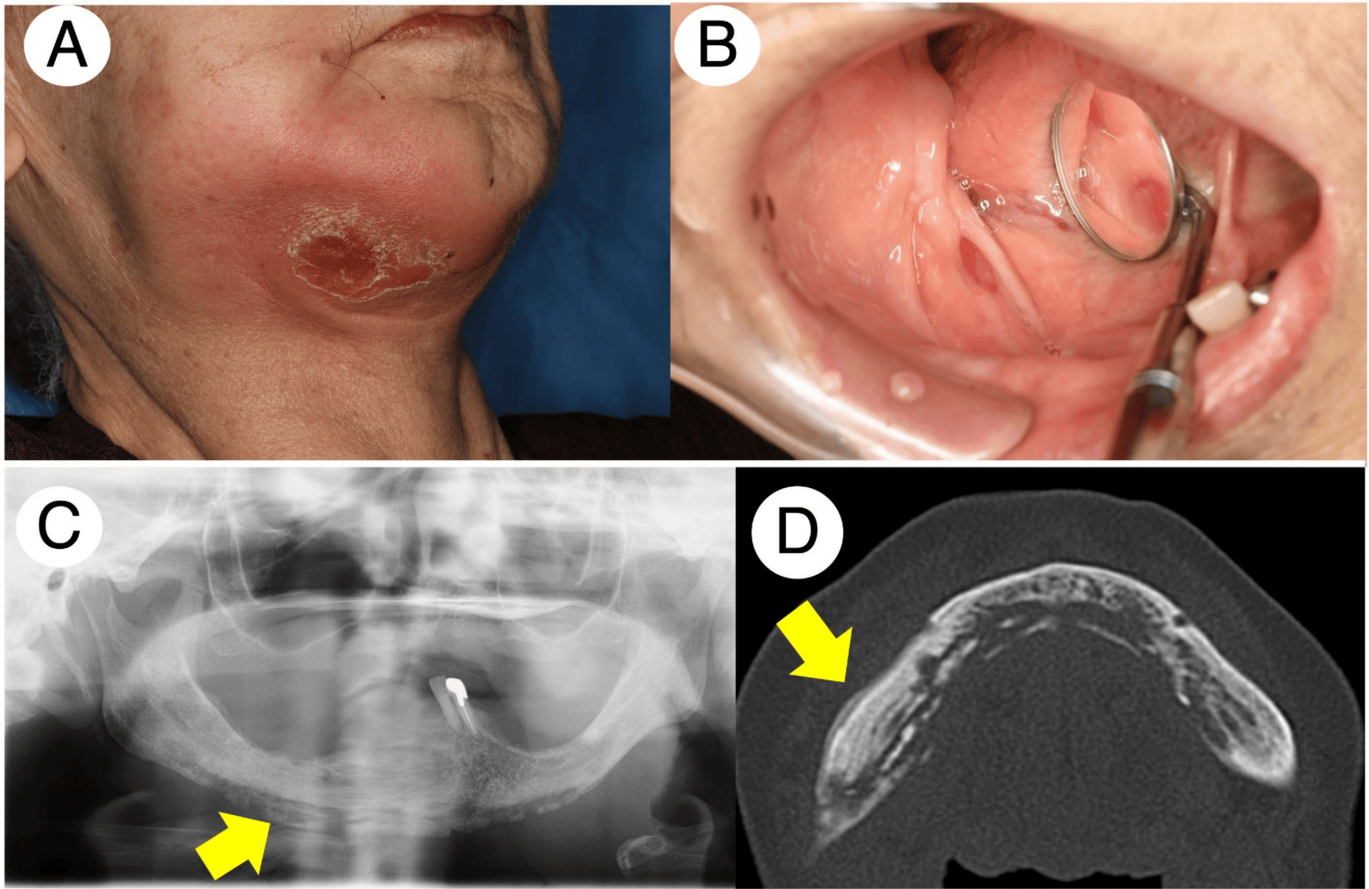
Patient 3 clinical and radiographic findings before the treatment. Extraoral examination found a significant swelling of the mandibular skin with redness (A). Intraoral examination identified abscess formation in the right-sided alveolar ridge with exposed bone probing through the fistula (B). Panoramic X-ray and MDCT exhibited evident signs of periosteal reaction throughout the mandibular edge (the yellow arrow indicates the finding of periosteal reaction) (C and D). MDCT: multidetector computed tomography

An 87-year-old female patient with a history of osteoporosis treated with BP for 19 months was referred to our department for a complaint of swelling in the right-sided mandibular and gingival swelling in the right-sided mandibular molar. The extraoral examination revealed swelling and apparent redness around the right-sided mandibular area and difficulty in opening her mouth (Figure 5A). Intraoral examination identified a fistula in the right-sided mandibular molar area with exposed bone probing through the fistula (Figure 5B). Panoramic X-ray examination revealed extensive osteolytic lesions with apparent periosteal reaction throughout the mandibular edge (Figure 5C). Furthermore, MDCT presented a periosteal reaction with sequestrum formation throughout the mandibular bone (Figure 5D). Consequently, the patient was diagnosed with osteonecrosis of the jaw suspicious of MRONJ stage 3 in the mandibular bone.

**Figure 6:**
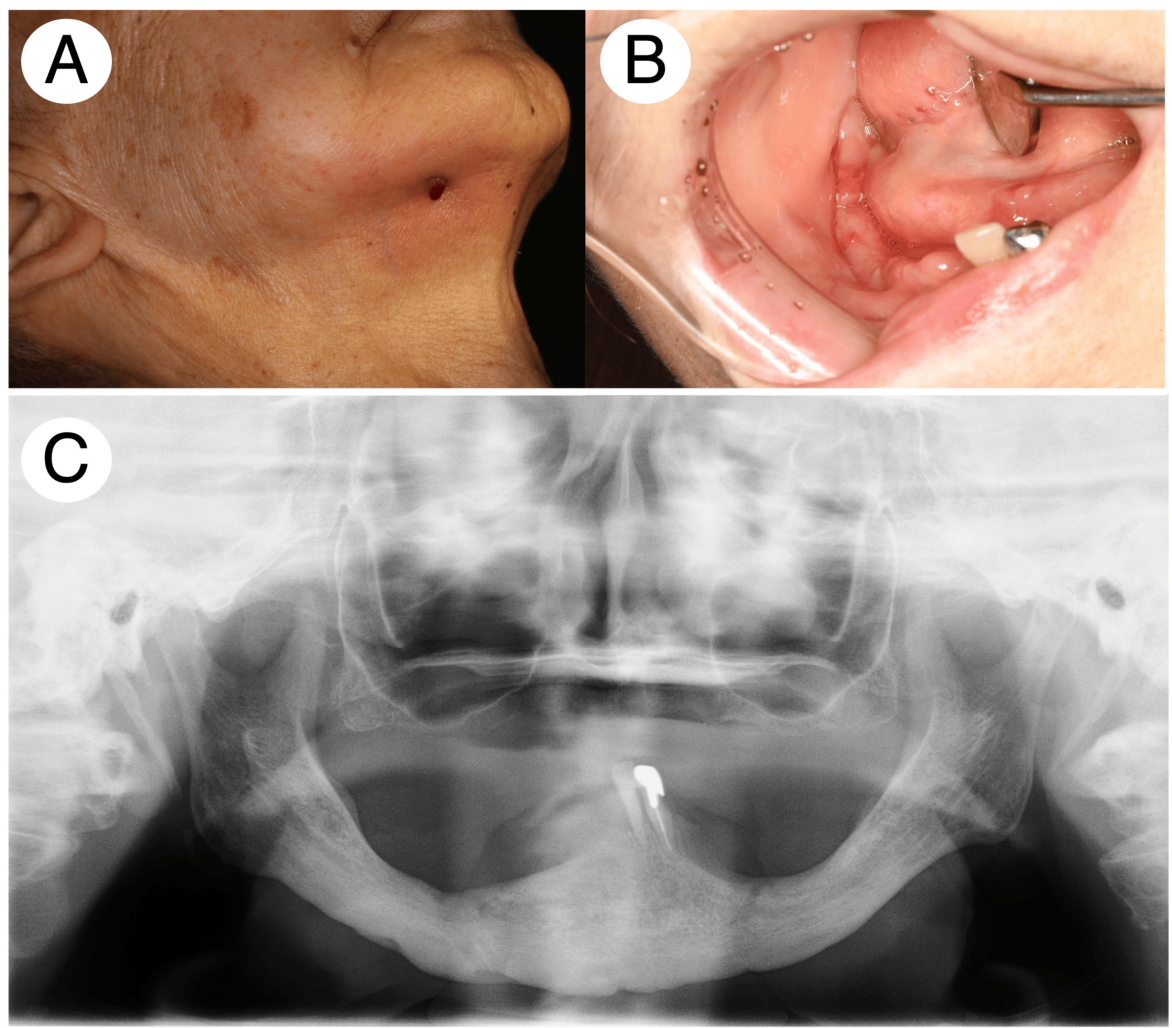
Patient 3 clinical and radiographic findings after the treatment. Extra- and intraoral examination detected the fistula covered with granulation tissue (A and B). Panoramic X-ray identified a mild increase in sclerotic changes in the mandibular bone without any signs of periosteal reaction (C).

The patient initially received oral irrigation regularly and antimicrobial treatment with AMPC 750 mg/day for 15 days in total until her hospitalization. Thereafter, the patient was hospitalized in our department and received HBO therapy 10 times. Owing to the apparent decline in her cognitive function during hospitalization, further HBO therapy was hampered. Twenty-four months after the treatment, an extraoral fistula remained, which was fully covered with granulation tissue, and the intraoral fistula disappeared in the affected area (Figure 6A, 6B). Panoramic X-ray revealed the disappearance of periosteal reaction and signs of bone regeneration throughout her mandibular bone (Figure 6C).

All the patients do not exhibit any acute symptoms after their treatment and are currently being monitored regularly.

## Discussion

Herein, we present a case series of older patients with advanced MRONJ that was well-controlled by the combination of conventional treatment and HBO therapy.

Thus far, HBO therapy has proven its efficacy in preventing and treating patients with ORN [11,13]. Although the effect of HBO therapy on patients with MRONJ remained unclear, our department has recently been managing patients with MRONJ by a systemic therapeutic strategy based on HBO therapy and evaluating them by nuclear medicine modalities. Previously, we demonstrated the validity of FDG-PET and bone SPECT on the diagnosis and evaluation of the effect of HBO therapy on patients with MRONJ [12]. Our authors found that FDG-PET showed a significantly higher SUVmax average on MRONJ lesions than on other types of osteomyelitis [12]. In addition, bone scintigraphy depicted a significant decrease in 99mTc in the MRONJ lesion in perfusion and static bone images after HBO therapy compared with that taken before HBO therapy, indicating a reduction in inflammation in patients with MRONJ. Therefore, HBO therapy would help improve the condition of patients with MRONJ, and this effect would be evaluated by nuclear medicine modalities [12].

Recently, a previous study revealed the validity of bone SPECT to assess the inflammatory activity of MRONJ quantitatively [14]. The authors developed a novel software, GI-BONE, which we also employed to assess the therapeutic effect on our patients. This software enables us to evaluate the inflammatory activity based on the SUVmax and MBV by comparing the indices between before and after MRONJ treatment [14]. Patients 1 and 2 showed a remarkable decrease in both SUVmax and MBV, indicating that our therapeutic strategy of combining HBO therapy provided certain benefits to improve the condition of patients with MRONJ. Thus, bone SPECT should be a useful tool to monitor MRONJ quantitatively.

In the management of patients who undergo HBO therapy, side effects generally associated with the therapy should be considered. Basically, in HBO therapy, patients are placed in a chamber where the pressure is 1.5-3.0 times greater than that of normal air. Thus, those who have lung disease, particularly untreated pneumothorax, and claustrophobia are not indicated for HBO therapy [15]. In addition, previous studies have reported the side effects of HBO therapy, including ear discomfort, barotrauma of the middle ear, sinus and paranasal sinus, and ocular complications such as myopic change [15,16]. Consequently, clinicians must pay attention to these potential side effects in managing patients who would undergo HBO therapy. Fortunately, all our patients did not experience any side effects throughout their treatment.

As for the mechanisms of HBO therapy, it may provide clinical benefits including (1) antimicrobial effect by the oxygenation of the affected tissue, resulting in the production of reactive oxygen species; (2) accelerating wound healing by promoting angiogenesis; and (3) improving blood circulation and hypoxia by decreasing gas volume resulting from edema under higher pressure [12,17]. That is, HBO therapy should help oxygen and antibiotics reach the MRONJ lesion by enhancing angiogenesis, resulting in healing. Moreover, a previous study suggested that patients with MRONJ who received HBO therapy exhibited a distinct bone healing process from normal bone remodeling, termed mini-modeling [12]. This unique process results from the regeneration of new bone above preexisting bones, shown as a smooth boundary between the preexisting bone and the regenerated bone. Because mini-modeling indicates osteoblast activity in the preexisting bone, the authors proposed mini-modeling as a predictive marker of the healing process among patients with MRONJ who underwent HBO therapy. Therefore, HBO therapy should promote healing in these mechanisms in patients with MRONJ.

Besides the effect of HBO therapy on patients, the patients received conventional treatment with oral irrigation and antimicrobial treatment with AMPC or AMPC/CVA as initial treatments. The number of antibiotics differed among patients considering their renal function; however, acute symptoms were relieved in each patient, proposing the need for initial treatment before HBO therapy. Moreover, each patient took an ARA drug holiday to prevent MRONJ progression. In the point of an ARA drug holiday in treating patients with MRONJ, previous studies have demonstrated that MRONJ treatment under an ARA drug holiday helps relieve MRONJ symptoms faster than treatment under continuing the drugs [4,18]. Furthermore, a recent study demonstrated that an ARA drug holiday was associated with better healing rates in MRONJ treatment than treatment under ARA continuation [19]. However, other studies have suggested that drug holidays did not influence the outcome of patients with MRONJ who underwent surgical treatment [4,20]. Moreover, a drug holiday may increase the risk of deteriorating bone resorption, resulting in fragility fractures [1]. Consequently, the necessity for drug holiday in managing patients with MRONJ remains to be unsubstantiated. In our cases, all patients had well-controlled systemic conditions; thus, we asked their family doctors to switch to active vitamin D metabolites instead of BP and/or anti-RANKL antibody.

Although this study presented a systematic therapeutic strategy based on HBO therapy, concluding that HBO therapy contributed to the improvement of the MRONJ condition is still difficult. Firstly, each patient’s treatment was initiated with oral irrigation and antimicrobial treatment before HBO therapy. Thus, it is difficult to conclude that the reduction in the inflammatory condition identified in nuclear medicine modalities was due to HBO therapy itself. Moreover, patients 1 and 2 experienced a distinct number of HBO therapy before conservative surgery. This was due to considering the patient’s decision and socioeconomic background. To evaluate the exact effect of HBO therapy on MRONJ, randomized controlled trials regarding the conventional treatment with/without HBO therapy for patients with MRONJ are necessary. However, this trial should be ethically difficult to investigate. Therefore, based on a previous study demonstrating the effectiveness of HBO therapy on patients with MRONJ, offering a minor invasive therapeutic strategy combined with conventional treatment with HBO therapy should be considered to manage those who struggle with this notorious inflammatory disease [12]. Secondly, patient 2 shows signs of inflammation in the partial area of the maxillary bone in the bone SPECT. While these findings indicated the remaining inflammatory condition, these findings would be reasonable to consider given that the timing of evaluating these modalities was one week after treatment, which would not be enough to relieve the inflammatory condition. Although panoramic X-ray and MDCT images did not detect any signs of further inflammation, these findings by bone SPECT should present important insight to detect early recurrence in further monitoring. Of course, not all patients with MRONJ are indicated for these modalities including MDCT and bone SPECT like patient 3, who did not agree to receive further examinations. Consequently, oral surgeons should consider the need to perform these examinations under regular monitoring based on patients’ symptoms and background.

## Conclusions

MRONJ is an intractable disease that sometimes requires extensive surgery. We experienced older patients with advanced MRONJ who were all well-controlled by conventional treatment with HBO therapy, which prevented them from undergoing extensive surgery. This case series presents that HBO therapy has the potential to be a therapeutic option as an adjuvant treatment. Furthermore, through our case series, bone SPECT would provide insights into evaluating the outcome of MRONJ treatment, highlighting the need for these modalities to monitor patients with MRNOJ.
